# Characterization of Basal Transcriptomes Identifies Potential Metabolic and Virulence-Associated Adaptations Among Diverse Nontyphoidal *Salmonella enterica* Serovars

**DOI:** 10.3389/fmicb.2021.730411

**Published:** 2021-10-13

**Authors:** Alexa R. Cohn, Renato H. Orsi, Laura M. Carroll, Ruixi Chen, Martin Wiedmann, Rachel A. Cheng

**Affiliations:** ^1^Department of Microbiology, Cornell University, Ithaca, NY, United States; ^2^Department of Food Science, Cornell University, Ithaca, NY, United States; ^3^Structural and Computational Biology Unit, European Molecular Biology Laboratory, Heidelberg, Germany

**Keywords:** transcriptomics, *Salmonella*, genomics, pathogen, virulence

## Abstract

The zoonotic pathogen *Salmonella enterica* includes >2,600 serovars, which differ in the range of hosts they infect and the severity of disease they cause. To further elucidate the mechanisms behind these differences, we performed transcriptomic comparisons of nontyphoidal *Salmonella* (NTS) serovars with the model for NTS pathogenesis, *S*. Typhimurium. Specifically, we used RNA-seq to characterize the understudied NTS serovars *S*. Javiana and *S*. Cerro, representing a serovar frequently attributed to human infection *via* contact with amphibians and reptiles, and a serovar primarily associated with cattle, respectively. Whole-genome sequence (WGS) data were utilized to ensure that strains characterized with RNA-seq were representative of their respective serovars. RNA extracted from representative strains of each serovar grown to late exponential phase in Luria-Bertani (LB) broth showed that transcript abundances of core genes were significantly higher (*p*<0.001) than those of accessory genes for all three serovars. Inter-serovar comparisons identified that transcript abundances of genes in *Salmonella* Pathogenicity Island (SPI) 1 were significantly higher in both *S*. Javiana and *S*. Typhimurium compared to *S*. Cerro. Together, our data highlight potential transcriptional mechanisms that may facilitate *S*. Cerro and *S*. Javiana survival in and adaptation to their respective hosts and impact their ability to cause disease in others. Furthermore, our analyses demonstrate the utility of omics approaches in advancing our understanding of the diversity of metabolic and virulence mechanisms of different NTS serovars.

## Introduction

Nontyphoidal *Salmonella* (NTS) contributes the greatest overall burden of foodborne disease worldwide, resulting in approximately 4.07 million Disability-Adjusted Life Years (DALYs), 93.8 million illnesses, and 155,000 deaths annually (Majowicz et al., 2010; Kirk et al., 2015). The genus *Salmonella* comprises two species [*S. enterica* and *S. bongori* (Desai et al., 2013; Worley et al., 2018)]; *S. enterica* is further divided into six recognized subspecies (*enterica*, *salamae*, *arizonae*, *diarizonae*, *houtenae*, and *indica*; Brenner et al., 2000) and three proposed novel subspecies (A, B, and C; Alikhan et al., 2018). There are currently 2,659 recognized serovars of *Salmonella* (Issenhuth-Jeanjean et al., 2014), many of which are associated with different host ranges and disease severities in affected hosts (Brenner et al., 2000). *S. enterica* subsp. *enterica* serovars (abbreviated hereafter as “*S*.”), which cause the majority of clinical infections, can be further divided into two broad categories: those that are host-adapted and those with broad host ranges.

Serovars are designated as having a broad host range if they can colonize and/or infect a wide range of hosts, as is the case for *S*. Javiana and *S*. Typhimurium, which have been isolated from a wide range of mammals, amphibians, reptiles, and birds (Iveson and Bradshaw, 1973; Uzzau et al., 2000; Rabsch et al., 2002; Srikantiah et al., 2004; Leahy et al., 2016). Conversely, serovars are designated as host-adapted if they preferentially infect one host, such as *S*. Dublin, which is host-adapted to cattle (Langridge et al., 2015; Harvey et al., 2017; Mohammed et al., 2017). Host-adapted serovars can be further classified as host-restricted if they are only capable of colonizing and or infecting one host (e.g., *S*. Typhi, which can only infect humans). Recent studies have identified genetic differences between serovars that cause gastrointestinal disease in a broad range of hosts (e.g., *S*. Typhimurium) and those that cause extraintestinal disease in a single host (e.g., *S*. Typhi). Host adaptation of extraintestinal *Salmonella* serovars is often associated with degradation of genes involved in anaerobic metabolism of inflammation-derived alternate terminal electron acceptors such as tetrathionate and nitrate, and sodium-galactoside transport (Nuccio and Bäumler, 2014; Langridge et al., 2015). However, little is known about potential host adaptations in serovars that are not associated with severe clinical illness in humans or animals, such as *S*. Cerro, which is frequently isolated from asymptomatic cattle, but is rarely associated with human clinical cases (Cummings et al., 2010; CDC, 2016, 2018b).

Despite ongoing control efforts, the incidence of human clinical cases of NTS has increased 33% in the United States since 2001 (CDC, 2018b). Furthermore, the distribution of serovars isolated from clinical cases has changed. In the United States, the five most common serovars isolated from human infections in 2016 were Enteritidis, Newport, Typhimurium, Javiana and I 4,[5],12:i:- (CDC, 2018b). Additionally, the U.S. Centers for Disease Control and Prevention (CDC) reported that from 2006 to 2016, *S*. Typhimurium incidence among human clinical salmonellosis cases decreased by 32.8% while *S*. Javiana incidence increased by 92.3% (CDC, 2018b), making *S*. Javiana a serovar of considerable public health concern. In contrast, *S*. Cerro, the most commonly reported serovar isolated from cattle without clinical signs in the United States, caused very few clinical human cases, with just 38 cases reported in 2016, compared to 4,581 and 2,719 cases reported for *S*. Typhimurium and Javiana, respectively (CDC, 2018b). While a few genomic studies have identified mutations in virulence factors that could explain the putative bovine host adaptation of *S*. Cerro (Rodriguez-Rivera et al., 2014; Kovac et al., 2017), it is still largely unknown why *S*. Cerro is rarely isolated from human clinical cases.

As a reflection of the appreciable diversity of *Salmonella*, NTS serovars vary in the number and type of virulence factors that they encode. For example, studies characterizing genomic differences among *S*. Cerro isolates have reported that many isolates of *S*. Cerro are missing multiple genes encoded in *Salmonella* pathogenicity islands (SPIs) 10, 12, and 13, as well as genes that may be involved in intestinal colonization (*orgA* and *phS*.) and genes involved in D-alanine transport (Rodriguez-Rivera et al., 2014; Kovac et al., 2017). In addition to differences in gene presence, differences in expression of virulence factors and other genes may also partially explain why serovars differ in host range and the type and severity of disease they cause in infected hosts. Thus, in addition to genomic analyses, comparative transcriptomic analyses can provide an enhanced understanding of virulence differences between *Salmonella* serovars. For example, a comparative transcriptomic study between serovar Enteritidis and Typhimurium strains with high- and low-infectivity showed that high-infectivity *S*. Typhimurium strains displayed higher transcript abundances of the SPI-1 type-3 secretion system (T3SS) genes compared to low-infectivity *S*. Typhimurium strains (Kolenda et al., 2021). Differences in SPI-1 T3SS transcript abundances were however not recapitulated between the high- and low-infectivity *S*. Enteritidis strains, highlighting possible differences in the virulence and virulence attenuation mechanisms between serovars. In contrast, Shah reported that low pathogenicity *S*. Enteritidis strains displayed lower transcript abundances of SPI-1 genes than high pathogenicity strains, suggesting that reduced SPI-1 transcription may be associated with reduced pathogenicity across different serotypes, even though not all strains classified as low pathogenicity may show reduced SPI-1 transcription (Shah, 2014). Overall, these studies further demonstrate the utility of transcriptomics in elucidating differences in virulence that are not readily apparent from genomic analyses alone.

In our study, we utilized comparative transcriptomic analyses to characterize *S*. Typhimurium, Javiana, and Cerro isolates to (i) expand our understanding of diverse NTS serovars representing different lineages of *Salmonella*, and (ii) gain new insight into adaptations that may have occurred in the understudied serovars Cerro and Javiana. Overall, our analyses identified multiple differences between the basal transcriptomes of *S*. Cerro, *S*. Javiana, and *S*. Typhimurium, particularly within virulence and metabolism associated genes and orthologous clusters, providing additional information on adaptations that may affect the ability of *Salmonella* to cause human infections.

## Materials and Methods

### Isolate Selection for Initial Phylogenetic and Genomic Analyses

Using metadata available on the NCBI Pathogen Detection database,^1^ we selected *S*. Typhimurium, Javiana, and Cerro genome sequences that represent the genetic and geographic diversity of isolates within each serovar. Selected isolates represented dates between 2014 and 2018 and a variety of geographic locations (see Supplementary File S1), and represented different NCBI SNP clusters (assuring representation of the currently characterized diversity associated with each of the three serotypes studied); isolates were selected to only represent genomes that were sequenced with the Illumina platform. Raw reads for selected isolates were downloaded from the NCBI Sequence Read Archive (SRA; https://www.ncbi.nlm.nih.gov/sra/; Leinonen et al., 2011). Preliminary analysis of WGS data downloaded from the NCBI Pathogen Detection Isolate Browser for an initial set of isolates revealed a polyphyletic structure in *S*. Cerro, with a smaller clade of five isolates clustering separately from the main *S*. Cerro clade within section Typhi (Supplementary Figure S1), which was consistent with previous findings that suggested that *S*. Cerro is a polyphyletic serovar (Worley et al., 2018). Therefore, only isolates belonging to the larger *S*. Cerro clade were included, as the smaller clade of *S*. Cerro is considerably more rare (Elnekave et al., 2020) and is not considered to be the predominant clade prevalent among cattle (Rodriguez-Rivera et al., 2014; Kovac et al., 2017). Additionally, WGS data for the three strains characterized with RNA-seq were included: *S*. Cerro FSL R8-2349 was sequenced at Cornell University Biotechnology Resource Center (Ithaca, NY), *S*. Javiana FSL S5-0395 was sequenced at Université Laval (Québec City, QC), and a closed genome of *S*. Typhimurium ATCC 14028S was downloaded from NCBI (Supplementary File S1).

### Draft Genome Assembly

Illumina adapters were trimmed using Trimmomatic v. 0.36 with default settings (Bolger et al., 2014). Sequence quality was determined using FastQC v 0.11.7 (Andrews, 2010) and genomes with fully-removed adapters were kept. Genomes were assembled with SPAdes v. 3.11.1 using the – careful option and *k*-mer sizes of 33, 55, 77, 99, and 127 (Bankevich et al., 2012). Qualities of draft genomes were assessed using QUAST v. 3.2 (Gurevich et al., 2013) and average coverage was calculated by mapping trimmed reads to their respective assemblies using BBMap v. 35.49 (Bushnell, 2014) and SAMtools v. 1.3.1 (Li et al., 2009). SISTR v. 1.0.2 was used to confirm the reported serotype (Yoshida et al., 2016). kSNP3 v. 3.1 was used to identify core SNPs among the isolates, including the three strains used in the RNA-seq analysis, using an optimal *k-*mer size of 19nt as determined using Kchooser (Gardner et al., 2015). RAxML v. 8.2.11 was used to construct a maximum likelihood phylogeny, based on core SNPs using the GTRGAMMA model (Tavarè, 1986; Yang, 1994) with the Lewis ascertainment bias correction (Lewis, 2001), and 1,000 bootstrap replicates (Stamatakis, 2006). Trees were visualized using Interactive Tree of Life v. 5 (Letunic and Bork, 2007).

### Genome Annotation, Identification of Orthologous Genes, and Assignment of Gene Ontology and Enzyme Commission Terms

Prokka v. 1.12 was used to annotate assembled draft genomes (Seemann, 2014). Roary v. 3.12.0 (Page et al., 2015) was used to identify pan genomes among isolates within each serovar individually, as well as for all isolates in all serovars combined and Scoary v. 1.6.14 was used to perform pan genome-wide association studies among serovars (e.g., *S*. Cerro vs. *S*. Javiana and *S*. Typhimurium) using the false discovery rate (FDR) method for multiple comparisons correction (Benjamini and Hochberg, 1995). A gene was defined as core to a serovar if it was detected in all isolates of that serovar (*n*=16 isolates per serovar) and a gene was defined as core to the *Salmonella* serovars studied here if it was detected in every isolate (*n*=48 total isolates). Gene ontology (GO) and enzyme commission (EC) terms were identified using Blast2GO v. 1.2.1 (Conesa et al., 2005) using protein coding sequences as input. Overrepresentation of GO and EC terms was determined by performing Fisher’s exact tests and calculating FDR-corrected *p*-values to control for false positives arising from multiple comparisons (Benjamini and Hochberg, 1995).

### Culturing Conditions

Stock cultures of *S*. Typhimurium ATCC 14028S, *S*. Cerro FSL R8-2349, and *S*. Javiana FSL S5-0395 were stored at −80°C in Luria-Bertani (Lennox; LB) broth (containing 5g NaCl/L, 5g yeast extract/L, and 10g tryptone/L) plus 15% glycerol. Cultures were streaked from glycerol stocks onto LB agar and incubated at 37°C for 18–22h to obtain single colonies. Individual colonies were inoculated into 5ml LB broth and incubated at 37°C with aeration (shaking at 200rpm) for 14–16h. Overnight cultures of each strain were then sub-cultured 1:1000 into side-arm flasks containing 50ml LB broth pre-heated to 37°C; these cultures were used for RNA collection for RNA-seq, after incubating cultures with shaking at 200rpm at 37°C until *Salmonella* reached late exponential phase (representing 4h post sub-culturing for all 3 strains; Supplementary Figure S2A). To collect and preserve cells for RNA extraction, 1ml of the late exponential phase *Salmonella* culture was added to 2ml of RNA Protect Bacteria reagent (Qiagen; Germantown, MD), followed by vortex agitation for 5s and incubation at room temperature for 5min. RNA isolation was performed on 3 independent replicate cultures for each strain.

### Phenol-Chloroform Extraction of Total RNA

Treated bacterial cells were collected by centrifugation at 3,220×*g* for 10min at 4°C. Supernatants were discarded, and pellets were stored at −20°C until RNA extraction. Bacterial pellets were resuspended in 200μl Tris-EDTA (TE) buffer containing 15mg/ml lysozyme and 100μg/ml proteinase K and were incubated at room temperature for 10min. Two milliliters of TRI Reagent were added to resuspended pellets (Zymo Research; Irvine, CA) and RNA was extracted by following standard protocols for phenol-chloroform extraction of nucleic acids (Sambrook and Russell, 2006).

### DNase I Treatment, Quality Assessment, Ribosomal RNA Depletion and Preparation of RNA Libraries

RNA was treated with Turbo DNase (ThermoFisher Ambion; Waltham, MA) as per manufacturer’s instructions, followed by phenol-chloroform extraction (Sambrook and Russell, 2006). SYBR Green Master Mix (ThermoFisher; Waltham, MA) was used for qPCR quantification of DNA contamination using primers that amplify a 150nt *rpoB* fragment (see Supplementary Table S1 for a list of primers). Additional rounds of DNase treatment were performed for samples with Ct values <33 (representing >50 copies of target DNA based on efficiency calculations for these primers). DNA-depleted RNA samples were assessed with the 6,000 RNA Nano chip and BioAnalyzer (Agilent Technologies; Santa Clara, CA) as per manufacturer’s instructions. Samples with five distinct peaks and an RNA integrity number >8 were used for RNA-seq analysis. The RiboZero Bacteria Kit (Illumina; San Diego, CA) was used to deplete samples of 16S and 23S rRNA as directed by manufacturer’s instructions. rRNA-depleted samples were then quantified using the Qubit 2.0 RNA High Sensitivity Kit (Thermo Fisher; Waltham, MA), and RNA-seq libraries were generated using the NEBNext Ultra II Directional RNA Library Prep Kit (New England Biolabs; Ipswich, MA) with 100ng rRNA-depleted RNA as input. Libraries were quantified with the Qubit 2.0 dsDNA High Sensitivity Kit (ThermoFisher; Waltham, MA) and the size distributions were determined with a Fragment Analyzer (Agilent Technologies; Santa Clara, CA).

### RNA Sequencing

Sequencing was performed at the Cornell University Biotechnology Resource Center (Ithaca, NY) using an Illumina NextSeq 500 sequencer with 75-nucleotide stranded single-end reads.

### RNA-Seq Data Analysis

Raw reads were pre-processed by trimming low quality and adapter sequences with cutadapt v. 1.8 using a minimum length of 50nt, a quality cutoff of 20, and the – match-read-wildcards option (Martin, 2011). Sequence reads were mapped to the *S*. Typhimurium 14028S, *S*. Javiana FSL S5-0395, and *S*. Cerro FSL R8-2349 genome assemblies using BWA v. 0.7.17 (Li and Durbin, 2009). The featureCounts function in the Rsubread v. 1.32.4 package (Liao et al., 2014) in R (R Development Core Team, 2010) was used to assign reads to genomic features (e.g., orthologous clusters). Outputs from featureCounts were used as input for pairwise comparisons of transcript abundances using variance modeling at the observational level (voom method) implemented in the R package limma v. 3.38.3 (Law et al., 2014; Ritchie et al., 2015). Raw counts were first converted to their log counts per million (logCPM) values and genes with <10 average read counts across the three replicates for a given serovar were filtered out. Read counts were normalized using the method of trimmed mean of M-values (TMM; Robinson and Oshlack, 2010). These normalization factors were subsequently used as a scaling factor for library sizes. Differences in transcript abundances were determined by fitting linear models to the normalized data. Comparisons of transcript abundances of genes, orthologous clusters, and GO terms were deemed significantly different if they had (i) FDR<0.05 and (ii) a |log_2_ fold change|>2. Significant differences in transcript abundances of GO terms were determined using goseq v. 1.34.1 (Young et al., 2010).

### Reverse Transcriptase Quantitative PCR

In order to confirm transcriptional differences, reverse transcriptase quantitative PCR (RT-qPCR) was performed for four genes (see Supplementary Table S1 for a full list of primer sequences). RNA isolation was performed as described above for RNA-seq and cDNA was prepared using the Superscript Reverse Transcription kit (ThermoFisher; Waltham, MA) according to manufacturer’s instructions. qPCR was performed using SYBR Green 2X Master Mix (Applied Biosystems; Foster City, CA) in a reaction containing 0.4μM each primer (see Supplementary Table S1) and 1μl cDNA (~15ng) as template. Fold expression was calculated using the ΔΔCt method as previously described (Livak and Schmittgen, 2001); results represent the averages of three independent experiments performed in technical duplicates. qPCR was performed on (i) the RNA collected for RNA-seq and (ii) independently collected RNA for *S*. Cerro FSL R8-2349 and *S*. Typhimurium ATCC 14028S. The LB was prepared using different batches of yeast extract (i.e., a different batch of yeast extract was used to prepare LB for growth of cultures for RNA-seq *vS*. qPCR confirmation as the bottle of yeast extract used to grow cultures for RNA-seq had been exhausted when we performed RT-qPCR).

### Data availability

RNA-seq data are available in the Gene Expression Omnibus (GEO) database under the BioProject ID PRJNA634123. Accession numbers of isolates used in this study are available in Supplementary File S1. Computational log files and scripts are available on GitHub (https://github.com/alexarcohn/salmonella_rnaseq).

## Results

### Preliminary Comparative Genomics Analyses to Facilitate Analyses and Interpretation of Transcriptomics Data

**Figure 1:**
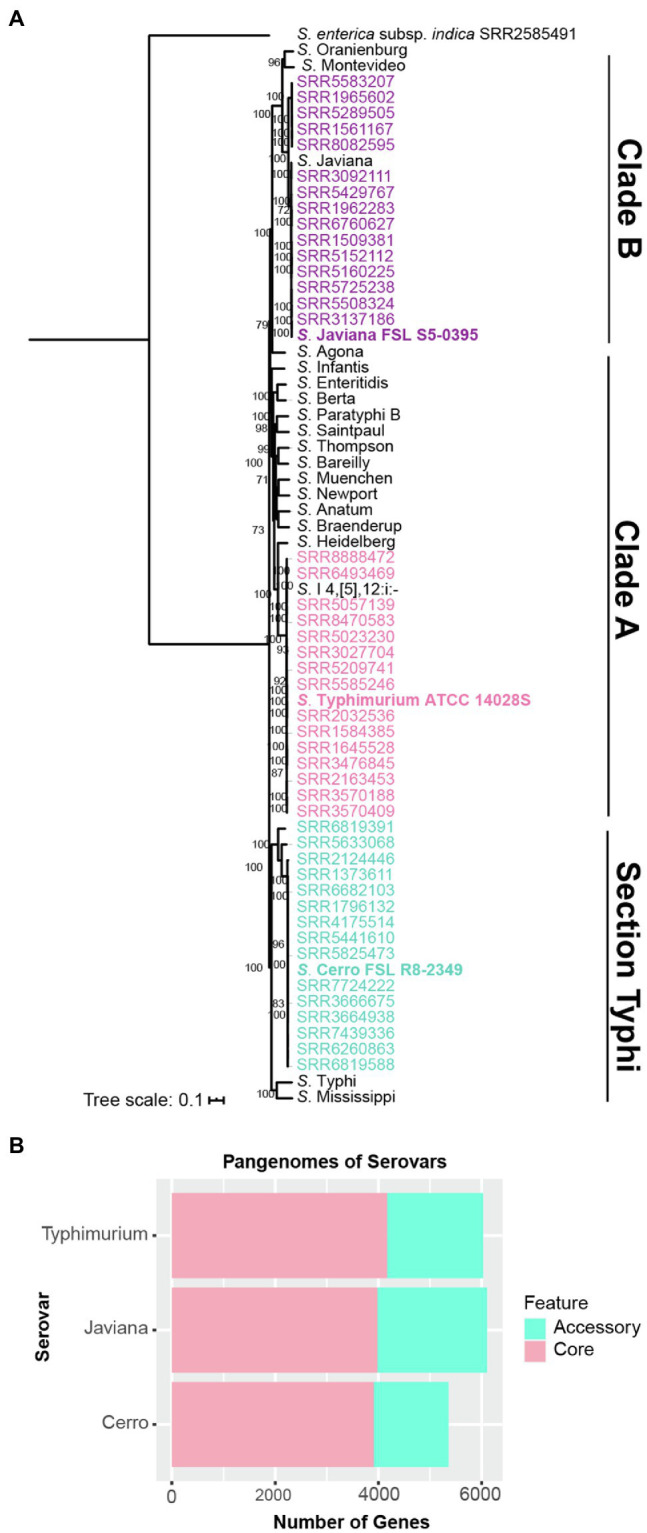
Genomic comparisons identify similar pan genome sizes for broad host range serovars Javiana and Typhimurium, but a smaller pan genome for cattle-associated serovar *S*. Cerro. **(A)** Maximum likelihood phylogenetic tree constructed from 55,425 core SNPs identified with kSNP3 for isolates representing NTS serovars Javiana (purple), Cerro (blue), Typhimurium (pink) and 18 additional serovars that represent the most common NTS serovars isolated from human clinical infections in the US; one additional assembly of *S*. Javiana (shown in black font) was included as this serovar is among the top four serovars isolated from human clinical infections in the US, however this isolate’s assembly was not included in the genomic analyses (i.e., core and pan genome size analyses; CDC, 2018b). Clade designations shown are based on those determined previously (Worley et al., 2018) for the serovars included in the tree. RAxML was used to infer the maximum likelihood tree utilizing a general time-reversible model with gamma-distributed sites and Lewis ascertainment bias correction; bootstrap values represent the average of 1,000 bootstrap repetitions and only bootstrap values >70 are shown. *S. enterica* subsp. *indica* SRR2585491 was used as an outgroup to root the phylogeny. Strains characterized by RNA-seq are shown in bold font. **(B)** Comparison of pan genome sizes for each serovar based on gene presence/absence analyses of 16 representative isolates per serovar.

Maximum likelihood phylogenetic analysis of 55,425 core SNPs among 48 isolates selected to represent the genetic, geographic and temporal diversity of isolates for serovars Cerro, Javiana and Typhimurium (16 isolates per serovar; see Materials and Methods, Supplementary File S1), along with representative isolates from 18 additional serovars that are frequently associated with human clinical illness in the US (CDC, 2018b), confirmed the phylogenetic classification of *S*. Javiana (clade B), *S*. Typhimurium (clade A), and *S*. Cerro isolates (section Typhi; Figure 1A; den Bakker et al., 2011; Worley et al., 2018). These analyses also confirmed that the isolates selected for RNA-seq cluster with other isolates of their respective serovar (Figure 1A). The isolates included in our analyses were selected from NCBI SNP clusters that comprised a total of 354 *S*. Cerro isolates, 4,846 *S*. Javiana isolates, and 10,066 *S*. Typhimurium isolates (accessed 01/25/2021; Supplementary File S1), supporting that the isolates used here for RNA-seq are appropriate representatives for these three serovars.

We also defined core and accessory genes to allow for comparisons of transcriptional patterns for core and accessory genes. Among the 48 isolates (16 isolates per serovar; see Supplementary Figure S3 for rarefaction curves for each serovar), *S*. Typhimurium isolates had the largest core genome (4,174 genes), followed by *S*. Javiana (3,985 genes) and *S*. Cerro (3,912 genes; Figure 1B). In contrast, *S*. Javiana isolates had the largest accessory genome (2,115 genes) compared to *S*. Typhimurium (1,854 genes) and *S*. Cerro (1,446 genes) isolates (Figure 1B). Both broad host range serovars, *S*. Typhimurium and *S*. Javiana, had similar pan genome sizes (6,028 and 6,100 genes, respectively), while the pan genome of *S*. Cerro was smaller (5,358 genes; Figure 1B). The *Salmonella* core and pan genomes for all 48 isolates in our study included 3,513 and 8,390 genes, respectively (Table 1; a complete list of gene presence/absence data can be found in Supplementary File S2); hence, transcript levels for specific genes could be compared among all three isolates for the 3,513 genes in the core genome. Among genes that were defined as core for two serovars (i.e., found in all 32 isolates for two serovars, but absent from all 16 isolates in the third serovar), 16 were core to *S*. Javiana and *S*. Cerro, 59 were core to *S*. Cerro and *S*. Typhimurium, and 103 were core to *S*. Javiana and *S*. Typhimurium (Table 1; Supplementary File S2).

**Table 1:** Core and accessory genome sizes among serovar Cerro, Javiana and Typhimurium isolates (*n*=16 per serovar).

Category*^a^*	Number of genes*^b^*
Core genes	3,513
Unique core Typhimurium genes	161
Unique core Javiana genes	105
Unique core Cerro genes	59
Genes core to Typhimurium and Javiana, but not Cerro	103
Genes core to Typhimurium and Cerro, but not Javiana	59
Genes core to Javiana and Cerro, but not Typhimurium	16
Number of genes in the pan genome	8,390

a Core genes represent genes that are present in every isolate for a given comparison.

b Gene presence/absence across isolates was determined with Roary (Page et al., 2015) and status as a core or accessory gene was determined using Scoary (Brynildsrud et al., 2016).

Additionally, we compared the gene content of strains selected to represent each serovar with the remaining 15 isolates of that serovar, to assess their suitability as being representative for the serovar. Genes that were uniquely present in the selected strain (i.e., were absent in all other isolates of that serovar) ranged from 11 in *S*. Cerro FSL R8-2349 to 148 in *S*. Javiana FSL S5-0395; *S*. Typhimurium ATCC 14028S contained 32 genes not found in any other *S*. Typhimurium isolate (Table 2). Further investigation of the 148 genes that were unique to *S*. Javiana FSL S5-0395 (compared to the other 15 *S*. Javiana isolates) revealed several type IV secretion system and *tra* genes (Supplementary File S2), suggesting that the high number of genes unique to this strain compared to other *S*. Javiana isolates may be the result of a mobile genetic element. Overall, these data support that the gene content of the strains selected for RNA-seq is similar to other isolates of the same serovar included in our study.

**Table 2:** Genome features of the three strains characterized with RNA-seq.

Strain	Genome size (Mbp)*^a^*	Number of contigs	G+C content (%)	Number of genes*^b^*	Number of genes unique to each strain*^c^*
*S*. Cerro FSL R8-2349	4.64	114	52.28	4,393	11
*S*. Javiana FSL S5-0395	4.68	28	52.22	4,444	148
*S*. Typhimurium ATCC 14028S	4.96	2	52.21	4,698	32

a Megabase pairs.

b Gene annotation and identification was performed using Prokka (Seemann, 2014).

c Unique genes were determined within serovar using Roary (Page et al., 2015).

We also performed GO term classification, which enabled binning of orthologous gene clusters, and subsequent enrichment analysis to identify pathways and processes that may be associated with a given serovar (Gene Ontology Consortium, 2004). GO term analyses revealed comparable numbers of GO terms identified among each serovar (range: 4,487–4,588 GO terms). The number of enzyme commission (EC) terms identified was also similar for each serovar and ranged from 955 to 980 EC terms (Supplementary File S3). *S*. Cerro had a total of 58 and 96 GO/EC terms significantly over- and underrepresented, respectively, as compared to *S*. Javiana (FDR<0.05; Supplementary File S3) as well as 64 and 102 GO/EC terms significantly over- and underrepresented, respectively, as compared to *S*. Typhimurium (FDR<0.05). Despite the fact that *S*. Javiana and *S*. Typhimurium are from different phylogenetic lineages (Worley et al., 2018), only four GO/EC terms were significantly overrepresented (FDR<0.05) among the *S*. Javiana isolates compared to the *S*. Typhimurium isolates (Supplementary File S3).

### On Average, Transcript Abundances of Core Genes Are Significantly Higher Than Transcript Abundances of Accessory Genes, Regardless of the Serovar

**Figure 2:**
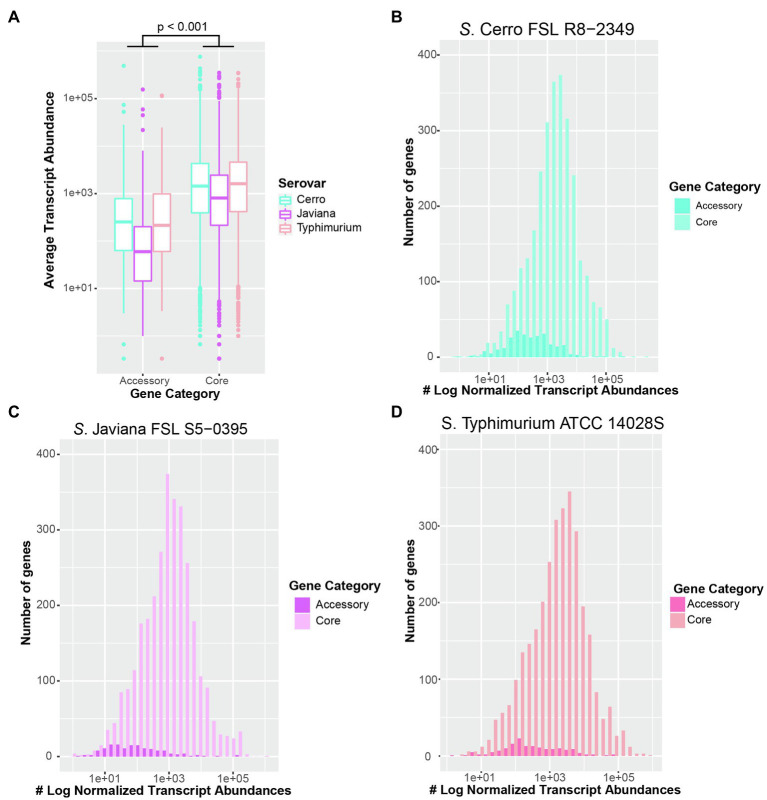
Transcript abundances of core genes are significantly higher than accessory genes in all three serovars. **(A)** Boxplot of average transcript abundances of core and accessory genes from three independent replicates of *S*. Cerro FSL R8-2349, Javiana FSL S5-0395, and Typhimurium ATCC 14028S cultured to late exponential phase in LB broth at 37°C. Significance was assessed with ANOVA after fitting a linear mixed-effects model to transcript abundances data and calculating the least square-means. Histograms of average transcript abundances of core (light colors) and accessory genes (dark colors) in **(B)** *S*. Cerro FSL R8-2349**, (C)** *S*. Javiana FSL S5-0395, and **(D)** *S*. Typhimurium ATCC 14028S. Results represent the average of three independent replicates. Core and accessory genes of 16 isolates per serovar were defined using Roary (Page et al., 2015).

The preliminary genomic analyses detailed above enabled us to use RNA-seq to (i) characterize the basal transcriptomes for strains representing two serovars whose transcriptomes have not been previously studied (*S*. Cerro and *S*. Javiana), and (ii) elucidate differences in transcriptome patterns between these two NTS serovars and *S*. Typhimurium. As Kröger et al. (2013) showed that growth of *S*. Typhimurium to late exponential phase in LB broth induced significantly higher expression of SPI-1, we collected and sequenced total RNA from cells grown to late exponential phase (Supplementary Figure S2A) in LB broth, to elucidate the basal transcriptome of representative strains for each serovar. For all three strains (Table 2), the average transcript abundances of previously defined core genes were significantly higher (*p*<0.001) than average transcript abundances of accessory genes (Figures 2A–D).

### Inter-Serovar Analyses Identify Several Genes With Significantly Higher and Lower Transcript Abundances

To further elucidate transcriptomic differences among serovars, we performed pairwise comparisons of transcript abundances for orthologous clusters that represent genes that were core to the two strains in each comparison (Supplementary Figure S2B). Orthologous clusters were defined as having significantly different transcript abundances if comparisons showed (i) an FDR<0.05 and (ii) log_2_ fold change |log_2_FC|>2. Overall, 294 (7.8%) orthologous clusters showed significantly different transcript abundances between *S*. Cerro FSL R8-2349 and *S*. Javiana FSL S5-0395, while 415 (10.8%) and 442 (11.3%) orthologous clusters showed significantly different transcript abundances for comparisons of *S*. Cerro FSL R8-2349 and *S*. Typhimurium ATCC 14028S, and *S*. Javiana FSL S5-0395 and *S*. Typhimurium ATCC 14028S, respectively (see Supplementary File S4 for a complete list). Clustering of transcript abundances of core genes suggested that the overall transcriptomes of *S*. Cerro FSL R8-2349 and *S*. Javiana FSL S5-0395 were more similar to each other, than they were to the transcriptome of *S*. Typhimurium ATCC 14028S (Supplementary Figure S4). Together, these results indicate that ~10% of orthologous genes have significantly different transcript abundances between serovars (under a single basal condition), and genetic relatedness may not be used to infer transcriptomic differences, supporting that transcriptomic methods provide an additional level of discrimination beyond that which can be achieved using genomic characterizations alone.

### Transcript Abundances of Central Carbon Metabolism Genes Are Higher in *S*. Typhimurium Than in *S*. Cerro and *S*. Javiana

**Figure 3:**
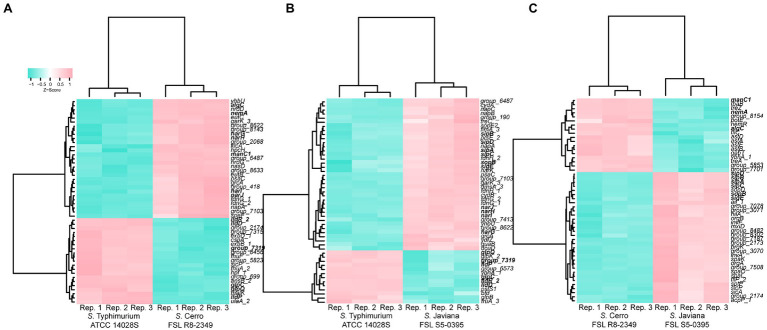
Comparison of transcript abundances in strains grown to late exponential phase in LB reveals many genes and orthologous clusters that are differentially expressed in inter-serovar comparisons. Heat map comparing the transcript abundances of the 50 most significantly differentially expressed (FDR<0.05, log_2_FC>|2|) orthologous clusters in **(A)** *S*. Typhimurium ATCC 14028S vs. *S*. Cerro FSL R8-2349**, (B)** *S*. Typhimurium ATCC 14028S vs. *S*. Javiana FSL S5-0395, and **(C)** *S*. Cerro FSL R8-2349 vs. *S*. Javiana FSL S5-0395. Differential expression was determined using variance modeling at the observational level (voom method), implemented in the R package limma. Transcript abundances from three biological replicates are shown (labeled as “Rep. 1–3” on the *x*-axis); *z*-scores represent the number of standard deviations that each log transformed transcript abundance for a given replicate differs from the average transcript abundances across all repetitions for that orthologous cluster. Clustering was performed using the Ward’s minimum variance method (Ward, 1963) based on Euclidean distance matrices. Genes are shown in bold if transcript abundances are significantly different in multiple comparisons (e.g., *sipA* shows significantly higher transcript abundances in *S*. Javiana compared to both *S*. Cerro and *S*. Typhimurium).

**Figure 4:**
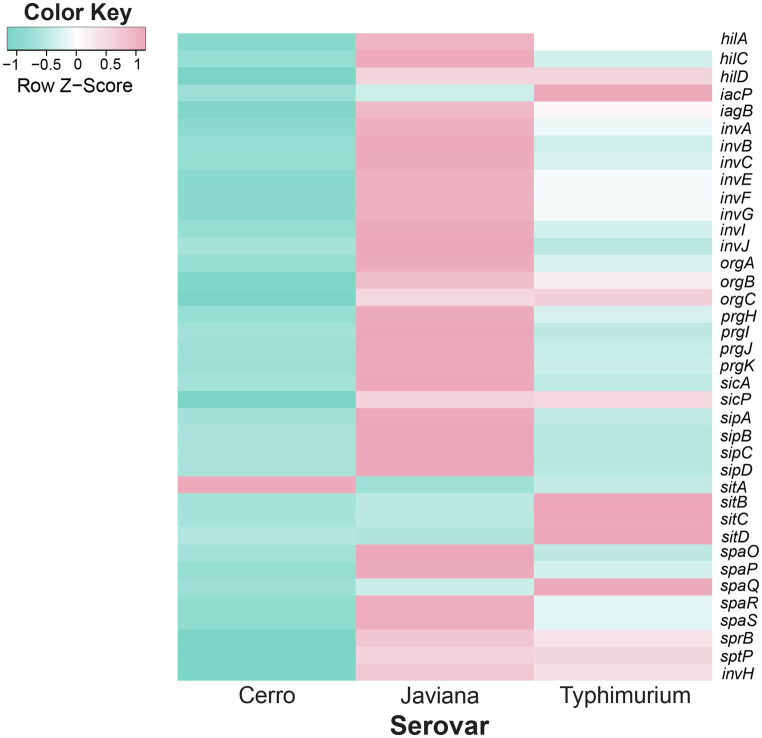
Inter-serovar comparisons demonstrate that SPI-1 transcript abundances are higher in serovars Javiana and Typhimurium compared to Cerro. Comparison of transcript abundances of genes in the SPI-1 locus. Transcript abundances were averaged from three biological replicates per strain (*S*. Cerro FSL R8-2349, *S*. Javiana FSL S5-0395, and *S*. Typhimurium ATCC 14028S). z-scores represent the number of standard deviations that a given strain’s transcript abundances differ from the transcript abundances across all strains for that gene.

Among 3,846 orthologous clusters present in both *S*. Cerro FSL R8-2349 and *S*. Typhimurium ATCC 14028S, 161 showed significantly higher transcript abundances in *S*. Typhimurium; these orthologous clusters included SPI-1 genes *hilA* and *sicP*, as well as eight *glp* genes and three *lld* genes, which are involved in glycerol degradation and L-lactate utilization, respectively (Figures 3A, 4; Supplementary File S4). By comparison, among the 3,895 orthologous clusters present in *S*. Javiana FSL S5-0395 and *S*. Typhimurium ATCC 14028S, 196 clusters had transcript abundances that were significantly higher in *S*. Typhimurium ATCC 14028S than in *S*. Javiana FSL S5-0395, including the same eight *glp* genes, and three *lld* genes that were also significantly higher in *S*. Typhimurium compared to *S*. Cerro (Figure 3B; Supplementary File S4). Compared to *S*. Cerro and *S*. Javiana, *S*. Typhimurium shows higher transcript abundances of genes involved in glycerol degradation and L-lactate utilization under the experimental conditions used here, suggesting that *S*. Typhimurium may better utilize glycerol and L-lactate under this condition when compared to the other two serovars.

### Transcript Abundances of Genes Involved in Metabolism Are Higher in *S*. Cerro Than *S*. Javiana and *S*. Typhimurium

Among the 294 orthologous clusters that showed significantly different transcript abundances between *S*. Cerro FSL R8-2349 and *S*. Javiana FSL S5-0395, 200 showed significantly higher transcript abundances in *S*. Cerro, including a number of genes involved in metabolic pathways such as genes involved in (i) nitrate assimilation (4 out of 5 genes in the *narUZYWV* operon), (ii) L-arginine degradation (5 out of 5 genes in the *astCADBE* operon), (iii) vitamin B12 biosynthesis (11 out of 25 genes in the *cob* operon), (iv) ethanolamine utilization (2 out of 17 genes in the *eut* operon), and (v) propionate metabolism (3 out of 4 genes in the *prpBCDE* operon; Figures 3C, 4; Supplementary File S4). Among the 415 orthologous clusters that had significantly different transcript abundances between *S*. Cerro FSL R8-2349 and *S*. Typhimurium ATCC 14028S, 254 showed significantly higher transcript abundances in *S*. Cerro (Supplementary File S4). Similar to the relationship observed for *S*. Cerro *vs. S*. Javiana, these 254 orthologous clusters also included genes involved in (i) nitrate assimilation (4 out of 5 genes in the *narUZYWV* operon), (ii) L-arginine degradation (1 out of 5 genes in the *astCADBE* operon), (iii) vitamin B12 biosynthesis (12 out of 25 genes in the *cob* operon), (iv) ethanolamine utilization (6 out of 17 genes in the *eut* operon), and (v) propionate metabolism (*pduC* and 3 out of 4 genes in the *prpBCDE* operon; Figure 3A; Supplementary File S4). These results suggest that under the conditions used in this experiment, *S*. Cerro shows higher transcript abundances of multiple metabolic processes compared to *S*. Javiana and *S*. Typhimurium and thus may more efficiently perform these processes under relevant conditions.

### Transcript Abundances of Invasion-Related Genes Are Higher in *S*. Javiana Than in *S*. Cerro and *S*. Typhimurium

Pairwise comparisons of the *S*. Javiana transcriptome with the transcriptomes of *S*. Cerro and *S*. Typhimurium identified 94 orthologous clusters with significantly higher transcript abundances in *S*. Javiana FSL S5-0395 compared to *S*. Cerro FSL R8-2349 (Figure 3C). These 94 orthologous clusters included 15 genes within SPI-1 (Figures 3C, 4; Supplementary File S4), which facilitate invasion of host epithelial cells (Lou et al., 2019). To confirm that the observed differences in SPI-1 gene transcript abundances were not due to mutations in known transcriptional regulators of SPI-1 [*barA*, *sirA*, *invF*, *rtS*., *hilA*, *hilC*, and *hilD* (Lou et al., 2019)] we aligned sequences of these genes from *S*. Cerro with *S*. Typhimurium and *S*. Javiana; these comparisons did not identify any nonsynonymous mutations or premature stop codons in *barA*, *sirA*, *invF*, *rtS*., *hilA*, *hilC*, and *hilD*. Other orthologous clusters with significantly higher transcript abundances in *S*. Javiana FSL S5-0395 compared to *S*. Cerro FSL R8-2349 included genes involved in histidine biosynthesis, sulfate transport, and trehalose catabolism (Supplementary File S4). In addition, 246 orthologous clusters showed significantly higher transcript abundances in *S*. Javiana FSL S5-0395 than *S*. Typhimurium ATCC 14028S (Figure 3B), including all 5 genes in the *narGHIJK* operon and 15 SPI-1 genes (Figures 3B, 4). These results suggest that like *S*. Cerro, the basal transcriptome of *S*. Javiana also shows key differences compared to *S*. Typhimurium, especially with regard to transcript abundances of SPI-1 genes.

### qPCR Highlights How Variability in LB Composition Can Impact Transcriptional Profiles

**Figure 5:**
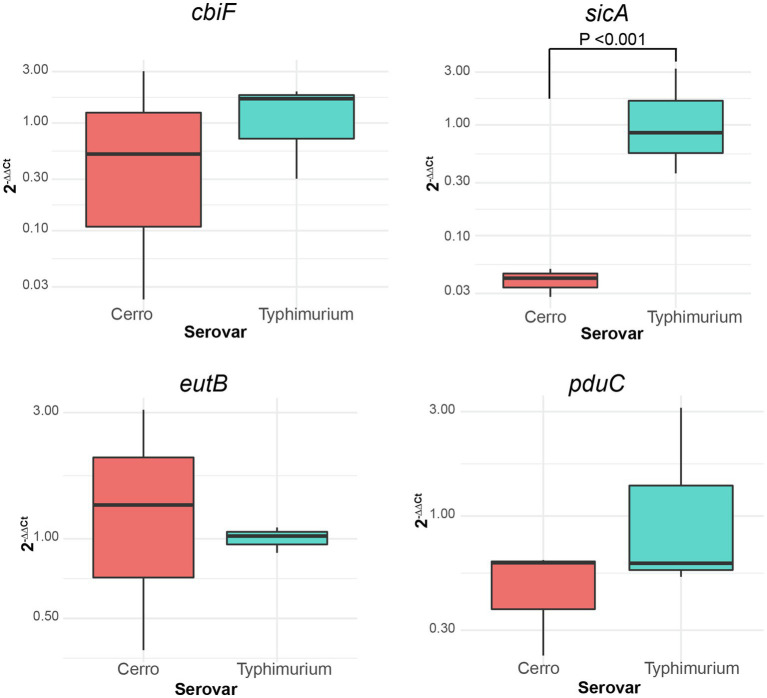
qPCR demonstrates how inherent variability in undefined media can impact transcript abundances of genes involved in metabolic functions. Boxplots comparing the relative expression (2^−∆∆Ct^) of *cbiF* (*cob* operon; involved in vitamin B12 biosynthesis functions), *eutB* (*eut* operon; ethanolamine utilization), *pduC* (*pdu* operon; 1,2-propanediol utilization), and *sicA* (SPI-1 locus; T3SS-mediated invasion) of *S*. Cerro FSL R8-2349 and *S*. Typhimurium ATCC 14028S normalized to the expression of *rpoB*. Results represent three independent replicates performed in technical duplicate. Significance was assessed with ANOVA after fitting a linear mixed effects model to the relative expression data and calculating least square means.

To probe the sensitivity of our RNA-seq findings to growth conditions, *S*. Cerro FSL R8-2349 and *S*. Typhimurium ATCC 14028S were grown (as three independent replicates) to late exponential phase in LB broth prepared with a different batch of yeast extract than that used for preparation of the LB broth utilized for the RNA-seq experiments; these cultures were used for RNA extraction and to perform RT-qPCR on four genes (*cbiF*, *eutB*, *pduC*, and *sicA*) that showed significantly different transcript abundances with RNA-seq. While we were able to recapitulate the finding that *sicA* transcript abundances are significantly higher in *S*. Typhimurium ATCC 14028S compared to *S*. Cerro FSL R8-2349, transcript abundances of *cbiF*, *eutB*, and *pduC* did not differ significantly between *S*. Cerro FSL R8-2349 and *S*. Typhimurium ATCC 14028S (Figure 5). However, when RT-qPCR for the same four genes was performed using cDNA produced from the RNA used for the RNA-seq experiments, we were able to reproduce the original RNA-seq data and found that transcript abundances of *cbiF*, *eutB*, and *pduC* were significantly higher in *S*. Cerro FSL R8-2349 compared to *S*. Typhimurium ATCC 14028S and that *sicA* transcript abundances were significantly higher in *S*. Typhimurium ATCC 14028S compared to *S*. Cerro. FSL R8-2349 These findings suggest that differences in LB composition can significantly impact gene expression.

## Discussion

*S*. Typhimurium has been extensively characterized, and remains the model for NTS (Garai et al., 2012). While numerous transcriptomic studies have been performed using *S*. Typhimurium (Kröger et al., 2012, 2013; Srikumar et al., 2015), such studies have only been performed for a handful of other NTS serovars including *S*. Newport (Dunn et al., 2020), *S*. Enteritidis (Deng et al., 2012; Shah et al., 2012; Shah, 2014; Kolenda et al., 2021) and *S*. Dublin (Huang et al., 2019). Here, we provide data for the basal transcriptomes of *S*. Cerro and *S*. Javiana, two serovars that have not been characterized by RNA-seq. We also demonstrate the utility of comparative transcriptomic analyses for identifying potential phenotypic differences among serovars that may be helpful for understanding niche adaptation of different serovars. Finally, we identified several metabolic pathways with higher transcript abundances in *S*. Cerro, and higher transcript abundances of SPI-1 genes in *S*. Javiana, compared to the model serovar *S*. Typhimurium, which may suggest distinct lifestyle adaptations in these understudied NTS serovars.

### Transcriptomic Approaches Provide Some Evidence for Virulence-Associated Differences Between NTS Serovars That Differ in Their Association With Human Infections

In our study, we show that growth in LB to late exponential phase resulted in higher transcript abundances of SPI-1 genes in strains representing *S*. Javiana as compared to *S*. Typhimurium; both of these serovars are frequently associated with human clinical salmonellosis. In addition, *S*. Cerro, a serovar that is considerably less common among human clinical salmonellosis cases, showed lower SPI-1 transcript abundances than either *S*. Javiana or *S*. Typhimurium. RT-qPCR analyses further confirmed that transcript abundances for *sicA*, a SPI-1 gene encoding a T3SS invasion protein chaperone (Fàbrega and Vila, 2013) that is indispensable for the invasion of epithelial cells (Lahiri et al., 2014), are lower in S. Cerro than *S*. Typhimurium, indicating the robustness of the RNA-seq findings. The importance of these findings is further supported by Kröger et al. (2013) who previously showed that growth of a different *S*. Typhimurium strain to late exponential phase in LB broth allowed for significant induction of SPI-1 gene transcription. The observed lower transcript abundances of SPI-1 genes in *S*. Cerro are also consistent with a previous finding that *S*. Cerro has a lower invasion capacity in cell culture than other NTS serovars, including *S*. Typhimurium (Rodriguez-Rivera et al., 2014), and may partially explain why *S*. Cerro is infrequently isolated from human clinical illnesses compared to *S*. Typhimurium (CDC, 2018a). The findings that *S*. Javiana showed higher transcript abundances of SPI-1 genes compared to both *S*. Typhimurium and *S*. Cerro represent an important new addition to our limited understanding of the biology of this serovar. Although an increasing prevalence of *S*. Javiana among human clinical infections in the US has been reported (Boore et al., 2015), only a few studies have explored the virulence of this organism. Our findings, combined with the fact that this serovar encodes both the typhoid and HlyE toxins (Mezal et al., 2014; Williams et al., 2014; Tamamura et al., 2017; which are not encoded in either Typhimurium or Cerro), suggest unique adaptations with regard to both virulence gene repertoire and virulence gene expression in this serovar; future studies examining the virulence potential of this serovar will be important and may facilitate a better understanding of virulence and transmission of *S*. Javiana as well as virulence gene regulation in *Salmonella* in general.

Additionally, to further elucidate mechanisms related to host adaptation and virulence, we assessed how transcript abundances of core and accessory genes within each serovar differ. While we found that on average, transcript abundances of core genes were significantly higher than transcript abundances of accessory genes, additional studies on the accessory genome transcriptome under multiple conditions may be beneficial for further elucidating the role(s) that these genes may play in virulence and niche adaptation. More specifically, characterization of transcript abundances of accessory genes may provide additional information on a serovar’s virulence and niche adaptation. For example, Chewapreecha et al. (2019) showed enrichment of virulence-related accessory genes among human clinical isolates and energy production and conversion-related accessory genes among environmental isolates of *Burkholderia pseudomallei*, potentially elucidating pathways of adaptation to multiple niches.

### Transcriptomic Analyses Suggest Metabolic Pathway Expression Patterns Consistent With the Infrequent Association of *S*. Cerro With Human Clinical Cases

RNA-seq data also indicated that transcript abundances of genes involved in metabolic processes, including vitamin B12 biosynthesis and ethanolamine and 1,2-propanediol utilization, may be higher in *S*. Cerro compared to *S*. Typhimurium and *S*. Javiana, at least under specific conditions. These findings were intriguing, considering that *S*. Cerro is frequently associated with cattle and rarely associated with human infections in the US, and could suggest unique adaptations in these pathways, which are linked to *Salmonella* survival and proliferation in the gastrointestinal tract (as discussed in more detail below). However, while RT-qPCR experiments confirmed these findings using the same RNA used for RNA-seq, when *S*. Cerro and *S*. Typhimurium were grown in LB prepared with different batches of yeast extract, we were unable to recapitulate the finding that transcript abundances of *cbiF* (a part of the *cob* operon, which encodes vitamin B12 biosynthesis functions), *eutB* (a part of the *eut* operon, which encodes ethanolamine utilization functions), and *pduC* (a part of the *pdu* operon, which encodes 1,2-propanediol utilization functions) were significantly higher in *S*. Cerro. Our inability to replicate our RNA-seq findings with RT-qPCR of cultures grown in a different batch of LB may be because LB is an undefined medium comprised of tryptone, yeast extract, and salt, and hence its composition of nutrients and vitamins can vary widely from batch to batch. Consistent with our findings, previous studies have identified differences in the presence of reactive oxygen species (Blair et al., 2013) and induction of SPI-1 (Sridhar and Steele-Mortimer, 2016) among cultures grown in commercial LB media obtained from different manufacturers. As such, we hypothesize that our RNA-seq results may represent differences that are observed under very specific and defined conditions. While our data suggest that the differences in transcription of vitamin B12 biosynthesis and ethanolamine and 1,2-propanediol utilization genes may only be detectable in certain batches of LB, we hypothesize that these differences also occur under infection-relevant conditions, which could include specific compartments within the intestinal tract.

Even though the differences in transcript abundances of vitamin B12 biosynthesis and ethanolamine and 1,2-propanediol utilization genes appear to be condition specific, combined with previous analyses of *S*. Cerro, our data still suggest a framework of how differences in regulation of ethanolamine and 1,2-propanediol play a role in the adaptation of *S*. Cerro to a distinct lifestyle. This is based on the fact that vitamin B12 is an essential cofactor for metabolism of ethanolamine and 1,2-propanediol (Jeter, 1990; Price-Carter et al., 2001). Metabolic changes such as utilization of 1,2-propanediol and ethanolamine have been shown previously to result in suppression of SPI-1 genes, which are located on a genomic island necessary for invasion of the epithelium (Lou et al., 2019). The down-regulation of SPI-1 genes occurs primarily *via* suppression of the SPI-1 transcriptional regulators HilA and HilD (Lostroh and Lee, 2001; Petrone et al., 2014); HilA is suppressed at the transcriptional level by 1,2-propanediol (Nakayama and Watanabe, 2006) and HilD is suppressed at the post-translational level by propionate (Hung et al., 2013). Consistent with these findings, our RNA-seq data showed significantly lower transcript abundances of *hilA* in *S*. Cerro compared to *S*. Javiana and *S*. Typhimurium. We also observed marginally lower, although not significantly lower, transcript abundances of *hilD* in *S*. Cerro; as HilD is regulated post-translationally we cannot exclude post-transcriptional downregulation of HilD (Hung et al., 2013). Importantly, previous studies have shown that *S*. Typhimurium displays stochastic expression of SPI-1 genes in the gut, with ~15% of the population upregulating transcription of SPI-1 genes (Ackermann et al., 2008), while the remaining population of *S*. Typhimurium instead preferentially performs vitamin B12-mediated metabolism of 1,2-propanediol and ethanolamine to propagate in the gut lumen (Ackermann et al., 2008; Sturm et al., 2011). With this model in *S*. Typhimurium in mind, significantly higher transcript abundances of genes associated with production of vitamin B12 for metabolism of ethanolamine and 1,2-propanediol and lower transcript abundances of SPI-1 in *S*. Cerro, could suggest that *S*. Cerro may follow a different pattern for survival in the gastrointestinal tract by preferentially proliferating in the gut lumen, rather than inducing inflammation *via* invasion of the gut epithelium, as has been documented for *S*. Typhimurium (Ackermann et al., 2008; Winter et al., 2010).

Taken together, the (i) previous observations that *S*. Cerro has a decreased ability to invade epithelial cells (Rodriguez-Rivera et al., 2014), (ii) the isolation of *S*. Cerro from cattle without clinical signs (Cummings et al., 2010; CDC, 2016), as well as the comparatively low number of human clinical cases reported for this serovar, and (iii) condition-specific significantly higher transcript abundances of genes involved in metabolism, such as biosynthesis of vitamin B12 and utilization of ethanolamine and 1,2 propanediol, collectively suggest that *S*. Cerro may activate alternate metabolic pathways to compete with the resident microbiota, rather than rely on inflammatory pathways to generate alternate electron acceptors as is the case for *S*. Typhimurium (Rivera-Chávez and Bäumler, 2015). While further characterization of the specific conditions that induce *S*. Cerro’s metabolic response and proliferation in the cattle digestive tract will be imperative for fully understanding and confirming *S*. Cerro’s apparent adaptation to cattle, we hypothesize that *S*. Cerro may use an alternative metabolic response under certain infection-relevant conditions within the gastrointestinal tract to compete with the resident cattle gut microbiota.

### Transcriptomic Comparisons Can Provide Broad Insight That Improve Our Understanding of *Salmonella* Virulence and Pathogenicity

In addition to our findings with regard to SPI-1 and transcription of metabolic functions associated with vitamin B12 biosynthesis as well as ethanolamine and 1,2-propanediol utilization genes, this study also provides insights on a number of other genes and transcriptional patterns. For example, the transcript abundances of genes in the AST pathway of L-arginine degradation (*astCADBE* operon) and nitrate reductase Z (*narUZYWV* operon) pathways were significantly higher in *S*. Cerro than both *S*. Javiana and *S*. Typhimurium. The higher transcript abundances found in these pathways in *S*. Cerro allows for generation of additional hypotheses on mechanisms that may enhance survival of *S*. Cerro under specific conditions in the gut. Experiments conducted in *E. coli* have shown that the AST pathway is necessary for the utilization of arginine and contributes to ornithine degradation (Schneider et al., 1998). Additionally, Lévi-Meyrueis et al. (2014) showed that wild-type *S*. Typhimurium displayed a higher competitive advantage during stationary phase than an *astA* mutant, suggesting that this pathway is important for survival of *Salmonella*. Higher transcript abundances of genes within the AST pathway in *S*. Cerro when grown to late exponential phase in LB may translate to a fitness advantage under specific conditions within the gut as increased metabolism of amino acids may provide increased nutrient availability, possibly supporting a more commensal lifestyle. Additionally, *S*. Cerro showed higher transcript abundances in genes in the *narUZYWV* operon compared to *S*. Javiana and *S*. Typhimurium. In *Salmonella*, this pathway of nitrate reduction is induced during exponential phase until the bacteria sense a lower availability of carbon sources consistent with the beginning of stationary phase (Torrez Lamberti et al., 2019). As such, the high transcript abundances within this operon in *S*. Cerro are most likely a result of the growth condition used in our study (growth in LB to late exponential phase). However, the ability to reduce nitrate also provides *Salmonella* with an advantage under specific conditions in the gut, as many obligate anaerobic bacteria, like those found in the gut microbiota, lack nitrate reductases and are thus unable to use nitrate as a terminal electron acceptor (Rivera-Chávez and Bäumler, 2015). As such, the significantly higher transcript abundances of the *narUZYWV* operon may provide *S*. Cerro with a selective advantage under certain conditions in the gut as increased transcription of a nitrate reduction mechanism may allow for increased survival due to the ability to utilize an alternate terminal electron acceptor. However, as with the higher transcript abundances observed in *S*. Cerro in the vitamin B12 biosynthesis and ethanolamine and 1,2-propanediol utilization, the higher transcript abundances observed in the *astCADBE* and *narUZYWV* operons in *S*. Cerro may only occur under specific infection-relevant conditions, and additional studies will be necessary to confirm this hypothesis.

In addition to transcript abundances that were higher in S. Cerro, we also identified 246 and 94 genes that showed high transcript levels in S. Javiana relative to either only S. Typhimurium or S. Cerro; the only genes with transcript abundances that were higher in S. Javiana relative to both S. Typhimurium and S. Cerro were the SPI-1 genes discussed above. One potentially interesting metabolic process with transcript abundances higher in S. Javiana compared to S. Typhimurium is nitrate reduction; specifically, the genes in the nitrate reductase A (*narGHI*) and periplasmic nitrate reductase (*napABC)* pathways showed higher transcript levels. As in *S*. Cerro, the higher transcription of these pathways in *S*. Javiana may help extend our understanding of this serovar’s lifestyle. The periplasmic nitrate reductase pathway has been shown to contribute to growth of *S*. Typhimurium in the host gut (Lopez et al., 2015) and its high transcription in *S*. Javiana may help its growth in the host intestine under specific conditions and hence may explain why it has become one of the most common serovars isolated from human clinical illnesses in the United States. As in *S*. Cerro, the observed higher transcript abundances of genes in the *narGHI* and *napABC* pathways in *S*. Javiana will need to be confirmed with follow up studies to understand how these adaptations may enhance fitness under certain conditions.

Additionally, we identified multiple pathways with significantly higher transcript abundances in *S*. Typhimurium compared to both *S*. Cerro and *S*. Javiana, including glycerol catabolism (*glp* operon) and L-lactate metabolism (*lld* operon). Interestingly, Kröger et al. (2013) found that both pathways were not highly expressed by *S*. Typhimurium strain 4/74 grown to late exponential phase in LB broth. The differences in expression of the glycerol catabolism and L-lactate metabolism pathways observed in *S*. Typhimurium strains ATCC 14028S (the strain used in this study) and 4/74 (the strain used by Kröger et al.) may be strain dependent and further emphasizes the importance of expanding the use of transcriptomic studies to multiple *Salmonella* serovars and strains within serovars, particularly since S. Typhimurium has been well described to represent considerable diversity, including possible host adapted strains (Rabsch et al., 2002; Branchu et al., 2018). Similar to the differences observed between *S*. Typhimurium strains 4/74 and ATCC 14028S, Shah (2014) and Kolenda et al. (2021) identified differences in expression of SPI-1 genes between low- and high-pathogenicity *S*. Enteritidis strains, highlighting the strain-level differences within serovars. Alternatively the differences between the results obtained here with strains ATCC 14028S and previously with strain 4/74 could be due to subtle differences in growth conditions, representing another reason why parallel transcriptomic studies with multiple strains will be important.

Overall, our study also illustrates the value of transcriptomic approaches to study differences among NTS serovars that may impact virulence and survival in the gut lumen. This is consistent with the fact that genomic and transcriptomic studies have been used previously for identifying how SNPs in non-coding regions influence host tropism (Langridge et al., 2015), virulence (Nuccio and Bäumler, 2014) and utilization of metabolic pathways (Seif et al., 2018). Similarly, other transcriptomic studies have been used to identify mechanisms of host adaptation for a variety of pathogens. For example, Kingsley et al. (2013) found significant differences in the expression of flagellar genes between host-restricted and broad host-range pathovars of *S*. Typhimurium grown at avian body temperature (42°C); this is likely important as downregulation of flagella at a host’s body temperature may aid in evading host immune responses. Similarly, Jalan et al. (2013) found multiple differences in the expression of virulence factors in two strains of *Xanthomonas citri* subsp. *citri* associated with broad and narrow host ranges. Taken together, the results provided in our study, as well as characterizations of other *Salmonella* serovars and other pathogens, highlight the utility of comparative transcriptomic studies in identifying key virulence differences between NTS serovars with broad or narrow host ranges.

It is important to note however that our study only examined the basal transcriptome for each serovar (i.e., one condition and one strain per serovar), which does not represent all conditions and stresses that *Salmonella* may experience while in a host. While comparisons of transcriptional responses of these serovars under infection-relevant conditions will be necessary for understanding how differences in gene regulation may impact differences in infection with different nontyphoidal *Salmonella*, our study provides important insight into the basal transcriptome of these serovars, and establishes methods for the comparison of transcript abundances of orthologous clusters that can be used in future studies that expand the number of strains, serovars, and culturing conditions to provide a more complete understanding of transcriptomic adaptations of different *Salmonella* serovars as well as insights why certain serovars may be associated with specific hosts, or why some serovars may be more likely to cause clinical disease than others. Our findings that some transcriptional differences could not be recapitulated across different LB batches, further illustrates the need for additional studies under different conditions.

## Data Availability Statement

RNA-seq data are available in the Gene Expression Omnibus (GEO) database under the BioProject ID PRJNA634123. Accession numbers of isolates used in this study are available in Supplementary File S1. Computational log files and scripts are available on GitHub (https://github.com/alexarcohn/salmonella_rnaseq).

## Author Contributions

RAC and MW designed the study. AC performed the experimental, computational, and data analyses. RO, LC, and RC helped with data analysis and edited and reviewed the final draft of the manuscript. AC, RAC, and MW co-wrote, edited, and reviewed the first draft of the manuscript. All authors contributed to the article and approved the submitted version.

## Funding

RAC was partially supported by USDA 2020-67034-31905.

## Conflict of Interest

The authors declare that the research was conducted in the absence of any commercial or financial relationships that could be construed as a potential conflict of interest.

## Publisher’s Note

All claims expressed in this article are solely those of the authors and do not necessarily represent those of their affiliated organizations, or those of the publisher, the editors and the reviewers. Any product that may be evaluated in this article, or claim that may be made by its manufacturer, is not guaranteed or endorsed by the publisher.

## Footnotes

^1^ https://www.ncbi.nlm.nih.gov/pathogens/

## Supplementary Material

Supplementary Figure S1 Initial phylogenetic analyses revealed a polyphyletic structure for *S*. Cerro. Maximum likelihood phylogenetic tree of *S*. Cerro (blue), *S*. Javiana (purple), and *S*. Typhimurium (pink) isolates constructed with core SNPs from 21 *S*. Cerro genomes, 16 *S*. Javiana genomes, and 16 *S*. Typhimurium genomes representing isolates with a range of isolation dates, sources, and locations, indicating polyphyly in *S*. Cerro. Isolates characterized by RNA-seq are shown in bold font. A general time-reversible model with gamma-distributed substitution sites was used for constructing the maximum likelihood tree in RAxML with a Lewis correction for ascertainment bias and 1,000 bootstrap repetitions; only bootstraps >70 are shown. The tree is rooted at midpoint.

Supplementary Figure S2 Comparison of core genes for strains characterized by RNA-seq following growth to late exponential phase in LB broth. (A) Overnight cultures of strains (*S*. Cerro FSL R8-2349, FSL *S*. Javiana FSL S5-0395 and *S*. Typhimurium ATCC 14028S) grown in LB broth, were sub-cultured 1:1000 into fresh LB broth; enumeration of colonies was performed each hour following sub-culturing. Growth assays were performed as three independent experiments; the variance in CFU/mL is captured by the error bars, which represent standard deviations of the mean. Samples were collected for RNA-seq at late exponential phase, approximately 4h after sub-culturing, as denoted with an arrow. (B) Venn diagram displaying the number of core genes among the three strains characterized by RNA-seq in our study. Gene presence/absence was determined using Roary (Page et al., 2015). A total of 3,565 genes are core to all three strains.

Supplementary Figure S3 Rarefaction curves of gene presence/absence data for isolates included for pan genome determination for each serovar. Rarefaction curves of the core and pan genomes of *S*. Cerro (*n*=16), *S*. Javiana (*n*=16), *S*. Typhimurium (*n*=16), and all isolates (*n*=48). A full list of isolates included in this analysis can be found in Supplementary File S1.

Supplementary Figure S4 Cluster analysis using a Euclidean distance matrix reveals that the transcriptomes of *S*. Cerro FSL R8-2349 and *S*. Javiana FSL S5-0395 are more similar to each other than to *S*. Typhimurium ATCC 14028S. A heat map of transcript abundances of all genes core to *S*. Cerro FSL R8-2349, *S*. Javiana FSL S5-0395, and *S*. Typhimurium ATCC 14028S (*n*=3,565), as determined with Roary (Page et al., 2015), across all three biological replicates (denoted as “Rep.”). Clustering was performed using the Ward’s minimum variance method (Ward, 1963) based on Euclidean distance matrices.

Supplementary Table S1 Primers used in our study.
