# Supplementary figures and images for: Characterization of Basal Transcriptomes Identifies Potential Metabolic and Virulence-Associated Adaptations Among Diverse Nontyphoidal Salmonella enterica Serovars
Source: Front Microbiol. 2021 Oct 13;12:730411. doi: 10.3389/fmicb.2021.730411 (PMC8552914; doi:10.3389/fmicb.2021.730411)

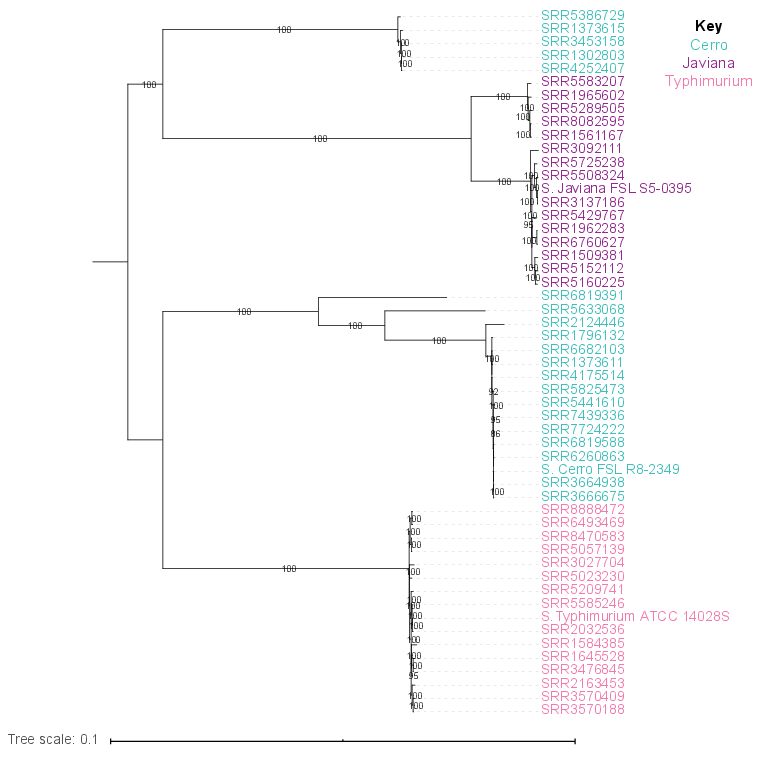

Supplement: Supplementary Figure S1 — Initial phylogenetic analyses revealed a polyphyletic structure for S. Cerro. Maximum likelihood phylogenetic tree of S. Cerro (blue), S. Javiana (purple), and S. Typhimurium (pink) isolates constructed with core SNPs from 21 S. Cerro genomes, 16 S. Javiana genomes, and 16 S. Typhimurium genomes representing isolates with a range of isolation dates, sources, and locations, indicating polyphyly in S. Cerro. Isolates characterized by RNA-seq are shown in bold font. A general time-reversible model with gamma-distributed substitution sites was used for constructing the maximum likelihood tree in RAxML with a Lewis correction for ascertainment bias and 1,000 bootstrap repetitions; only bootstraps >70 are shown. The tree is rooted at midpoint. [file Image_1.JPEG]

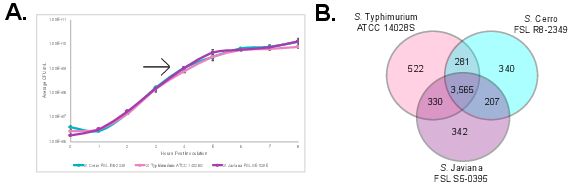

Supplement: Supplementary Figure S2 — Comparison of core genes for strains characterized by RNA-seq following growth to late exponential phase in LB broth. (A) Overnight cultures of strains (S. Cerro FSL R8-2349, FSL S. Javiana FSL S5-0395 and S. Typhimurium ATCC 14028S) grown in LB broth, were sub-cultured 1:1000 into fresh LB broth; enumeration of colonies was performed each hour following sub-culturing. Growth assays were performed as three independent experiments; the variance in CFU/mL is captured by the error bars, which represent standard deviations of the mean. Samples were collected for RNA-seq at late exponential phase, approximately 4h after sub-culturing, as denoted with an arrow. (B) Venn diagram displaying the number of core genes among the three strains characterized by RNA-seq in our study. Gene presence/absence was determined using Roary (Page et al., 2015). A total of 3,565 genes are core to all three strains. [file Image_2.JPEG]

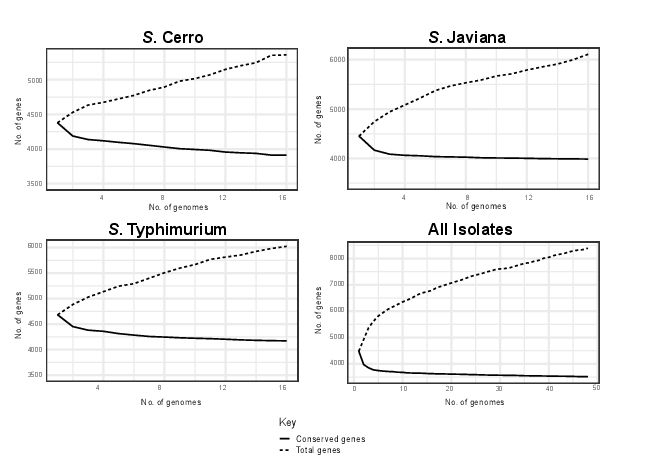

Supplement: Supplementary Figure S3 — Rarefaction curves of gene presence/absence data for isolates included for pan genome determination for each serovar. Rarefaction curves of the core and pan genomes of S. Cerro (n=16), S. Javiana (n=16), S. Typhimurium (n=16), and all isolates (n=48). A full list of isolates included in this analysis can be found in Supplementary File S1. [file Image_3.JPEG]

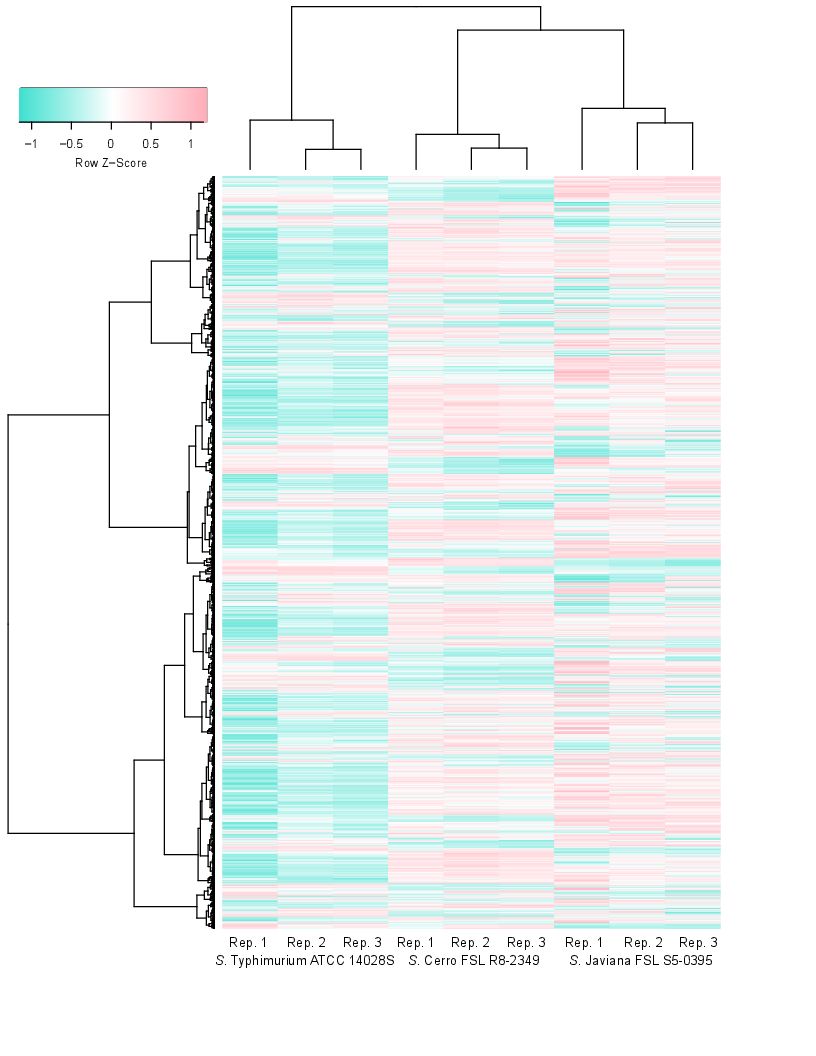

Supplement: Supplementary Figure S4 — Cluster analysis using a Euclidean distance matrix reveals that the transcriptomes of S. Cerro FSL R8-2349 and S. Javiana FSL S5-0395 are more similar to each other than to S. Typhimurium ATCC 14028S. A heat map of transcript abundances of all genes core to S. Cerro FSL R8-2349, S. Javiana FSL S5-0395, and S. Typhimurium ATCC 14028S (n=3,565), as determined with Roary (Page et al., 2015), across all three biological replicates (denoted as “Rep.”). Clustering was performed using the Ward’s minimum variance method (Ward, 1963) based on Euclidean distance matrices. [file Image_4.JPEG]
