# Supplementary material for: Characterization of Basal Transcriptomes Identifies Potential Metabolic and Virulence-Associated Adaptations Among Diverse Nontyphoidal Salmonella enterica Serovars
Source: Front Microbiol. 2021 Oct 13;12:730411. doi: 10.3389/fmicb.2021.730411 (PMC8552914; doi:10.3389/fmicb.2021.730411)
Supplement: Supplementary Table S1 — Primers used in our study. [file Table_1.docx]

**Table S1.** Primers used in our study.

| **Primer Name** | **Target** | **Primer Sequence (5’ to 3’)** |
| --- | --- | --- |
| RM85 | *rpoB*^1^ | TACGGGACGCATCCACGTAC |
| RM86 | *rpoB*^1^ | GTGCGAACATGCAACGTCAG |
| AC05 | *cbiF* | GATCCACGCTGTGTATGGTTC |
| AC06 | *cbiF* | CAGTAATCCAGCAGTTCGGTA |
| AC13 | *sicA* | CAGCTTCATCCAGTCGTCCCT |
| AC14 | *sicA* | CAGCGAAGAACGTGTTGCGGA |
| AC25 | *eutB* | TGACCGATGACGTGGAGAAC |
| AC26 | *eutB* | GCCCTGGGTCGGAATATTGAA |
| AC27 | *pduC* | TTTCCGTCCAGCTCGGTTAC |
| AC28 | *pduC* | TTTGAAGCACTGGCGAAACG |
| AC21 | *rpoB*^2^ | GAGTACATCGCAGGTAAAG |
| AC22 | *rpoB*^2^ | GAAACGCTGTTTACCAACG |

^1^RM85 and RM86 were utilized to check for genomic DNA depletion and were optimized for qPCR in a previous study.

^2^AC21 and AC22 were used for qPCR in Cerro and Typhimurium and were optimized for the strains used in this study.
